# Assessment of the Acute Phase Response in Healthy and Injured Southern White Rhinoceros (C*eratotherium simum simum*)

**DOI:** 10.3389/fvets.2019.00475

**Published:** 2020-01-09

**Authors:** Emma H. Hooijberg, Carolyn Cray, Gerhard Steenkamp, Peter Buss, Amelia Goddard, Michele Miller

**Affiliations:** ^1^Department of Companion Animal Clinical Studies & Centre for Veterinary Wildlife Studies, Faculty of Veterinary Science, University of Pretoria, Pretoria, South Africa; ^2^Department of Pathology & Laboratory Medicine, Miller School of Medicine, University of Miami, Miami, FL, United States; ^3^Veterinary Wildlife Services, South African National Parks, Kruger National Park, Skukuza, South Africa; ^4^Division of Molecular Biology and Human Genetics, Department of Science and Technology-National Research Foundation Centre of Excellence for Biomedical TB Research, Faculty of Medicine and Health Sciences, Medical Research Council Centre for Tuberculosis Research, Stellenbosch University, Stellenbosch, South Africa

**Keywords:** acute phase, *Ceratotherium simum*, fibrinogen, haptoglobin, iron, SAA

## Abstract

Acute phase reactants (APRs) have not been investigated in white rhinoceros (*Ceratotherium simum*). This study aimed to identify clinically useful APRs in this species. Reference intervals (RIs) were generated for albumin, fibrinogen, haptoglobin, iron and serum amyloid A (SAA) from 48 free-ranging animals, except for SAA (*n* = 23). APR concentrations between healthy animals and those with tissue injury (inflammation) (*n* = 30) were compared. Diagnostic performance was evaluated using receiver-operator characteristic (ROC) curve and logistic regression analyses. RIs were: albumin 18–31 g/L, fibrinogen 1.7–2.9 g/L, haptoglobin 1.0–4.3 g/L, iron 9.7–35.0 μmol/L, SAA <20 mg/L. Iron and albumin were lower and fibrinogen, haptoglobin and SAA higher in injured vs. healthy animals. Iron showed the best diagnostic accuracy followed by fibrinogen, albumin, haptoglobin and SAA. Iron ≤ 15.1 μmol/L and haptoglobin >4.7 g/L were significant predictors of inflammatory status and together correctly predicted the clinical status of 91% of cases. SAA > 20 mg/L had a specificity of 100%. In conclusion, albumin and iron are negative and fibrinogen, haptoglobin and SAA positive APRs in the white rhinoceros. The combination of iron and haptoglobin had an excellent diagnostic accuracy for detecting inflammation.

## Introduction

The acute phase reaction is a systemic innate immune response to infection, inflammation, injury and stress (1). Acute phase reactants (APRs) are substances [mostly acute phase proteins (APPs)] that either increase (positive APR) or decrease (negative APR) in the blood during the acute phase reaction (2). Measurement of APRs is commonly used in domestic species to detect and monitor the course of inflammatory disease, to prognosticate and to evaluate health on a herd basis (1–3). Commonly measured APRs include albumin (negative APP; all species), C-reactive protein (CRP) (positive APP; dog), serum amyloid A (SAA) (positive APP; dog, cat, horse, cow), haptoglobin (positive APP; dog, horse, cow), fibrinogen (positive APP; all species), and iron (negative APR; horse) (1–4). APRs have also been shown to be useful for these purposes in non-domestic mammalian species (5–17). For example, SAA and haptoglobin were increased in rhesus macaques with chronic active inflammation, in clinically abnormal zebras, in diseased Florida manatees, and in Asian elephants with pododermatitis, while SAA was increased in two black rhinoceros with ulcerative dermatitis (5, 6, 8, 13, 15). Furthermore, decreased serum iron has been shown to be a reliable indicator for inflammation in horses, a member of the same order of Perissodactyla as the white rhinoceros (4, 18, 19).

The southern white rhinoceros (*Ceratotherium simum simum*) (hereafter referred to as the white rhinoceros) is currently heavily threatened by poaching across the range states where it occurs. More than 5,500 animals have been killed by poachers in South Africa since 2013 (20). Many rhinoceros actually survive poaching attempts but need veterinary care for their injuries. Rhinoceros calves injured or orphaned due to poaching activity need to be cared for in rehabilitation centers (21). Several reports describe the clinical pathology of this species in order to better understand, diagnose and treat these animals (22–24). One significant finding was that injured white rhinoceros did not exhibit serum protein electrophoretic changes consistent with inflammation, but showed decreases in various protein fractions that were probably related to increased protein loss and catabolism during wound healing (22). The expected acute phase response appeared to be masked by these protein changes, and targeted measurement of specific APRs would be informative.

The objectives of this study were therefore to generate reference intervals (RIs) for selected APRs in the white rhinoceros and to investigate the acute phase response and diagnostic utility of APRs in animals with tissue injuries.

## Materials and Methods

### Study Population and Sampling

The group of healthy animals used as the reference sample group consisted of 50 free-ranging white rhinoceros from the Kruger National Park, South Africa (23°49′60″S, 31°30′0″E). Only adult animals were included in the reference sample group. White rhinoceros were considered to be healthy based on normal physical examination findings, completed while rhinoceros were immobilized. Horn length and body size were used to determine age (adults > 7.0 years) (25, 26). Calves, subadults, and any adult white rhinoceros exhibiting bullet or dehorning wounds, or any other visible abnormalities were excluded from this reference sample group.

The rhinoceros were immobilized primarily for translocation and other management purposes. Immobilization was performed according to the South African National Parks Animal Use and Care Committee approved Standard Operating Procedure for the Capture, Transport and Maintenance in Holding Facilities of Wildlife. The immobilization protocol used for these animals has been fully described elsewhere (23) Blood samples were collected within 15 min of immobilization, directly from the auricular vein into serum and sodium citrate vacuum collection tubes (Greiner Bio-One, Lasec S.A., PTY LTD Cape Town, 7405, South Africa), which were placed upright to clot (serum) in a cooler box. Sample tubes were centrifuged within 3 h of collection at 1,300 g for 10 min. Aliquoted serum and citrated plasma were frozen for up to 22 months at −80°C until analysis was performed.

Samples from a group of 30 white rhinoceros of diverse ages with tissue trauma of various severities and chronicities were used to represent animals with inflammation (22). This group was further subdivided into animals with either acute (duration of injury ≤ 2 days) or chronic injuries, when this information was available in clinical records. Of these animals, 23, including two calves, were from the Kruger National Park, and samples were collected using the protocol described above. These samples were frozen at −80°C for 6–27 months. Four samples originated from another research project investigating injured white rhinoceros and were stored at −80°C in the clinical pathology laboratory of the Onderstepoort Veterinary Academic Hospital (OVAH). Immobilization and sampling protocols for these four individuals were not known. Samples from three white rhinoceros calves that were inpatients in the OVAH were also included and were collected, without immobilization, from the auricular vein. For these last seven individuals, serum was received by the laboratory in serum vacuum tubes which were left to clot for 30 min and centrifuged at 2,100 g for 8 min. The serum was aliquoted and frozen at −20°C for 6–8 months for the calves, and at −80°C for 28–36 months for the other four adults.

Batches of samples were left to thaw at room temperature before analysis, then mixed and centrifuged at 2,100 g for 8 min.

### Sample Analysis and Assay Performance

All assays, apart from SAA and fibrinogen, were performed using a wet chemistry analyzer, the Cobas Integra 400 Plus (Roche Products (Pty) Ltd, Basel, Switzerland) as per manufacturer's instructions. Methods and standards were as follows: albumin, bromocresol green with a modified human serum calibrator [Roche Products (Pty) Ltd, Basel, Switzerland]; iron, ferrozine zinc with a modified human serum calibrator [Roche Products (Pty) Ltd, Basel, Switzerland]; haptoglobin, colorimetric peroxidase assay with a modified serum calibrator (species unknown) (PHASE Haptoglobin Assay, Tridelta, Maynooth, Ireland). Fibrinogen was determined using the modified Clauss method with a modified human plasma calibrator on an ACL Elite (Instrumentation Laboratory, Munich, Germany). SAA was measured using a multispecies sandwich ELISA (PHASE SAA, Tridelta, Maynooth, Ireland) using the bovine calibration and dilution protocol, according to the manufacturer's instructions. For all methods, apart from haptoglobin and SAA, assay performance [bias, imprecision (CV_I_) and total analytical error] was monitored by daily internal quality control procedures according to laboratory protocols and performance goals published for veterinary species (27, 28). Samples were analyzed once, apart from SAA where duplicates were always measured, and a repeat measurement of haptoglobin in seven samples from the injured group.

Partial analytical validation was carried out for haptoglobin and SAA. Two pools of sera were created, one from the healthy group with expected low concentrations of APP and one from the injured group with expected high concentrations. For haptoglobin, intra-assay imprecision and linearity were determined using recommended protocols as previously described; inter-assay imprecision measurements were limited to 18 measurements over 13 days due to expiration of kits (24, 29). The performance goal for maximum imprecision (CV_MAX_) was set at 8.5% (28). For SAA, intra-assay imprecision was calculated from the root mean square of duplicate measurements (30). The limit of quantification (LoQ) of the ELISA for SAA in white rhinoceros was determined using a regression trend analysis and determined to be the SAA concentration at which imprecision was 20% (31). This imprecision goal was selected and deemed reasonable based on the experience of the authors (EHH and CC), given the lack of published performance goals for SAA in veterinary species. Linearity for the SAA assay was evaluated using serial dilutions of the high concentration pool. Linearity for haptoglobin and SAA was determined using Spearman's correlation coefficient (*r*) and linear regression analysis.

### Data Analysis

#### Reference Intervals (RI)

The procedure for RI generation followed the American Society for Veterinary Clinical Pathology (ASVCP) guidelines using Reference Value Advisor version 2.1 (32, 33). Initial data analyses included descriptive statistics, visual inspection of histograms, outlier identification with Tukey and Dixon tests, evaluation of normality with the Anderson-Darling test, and evaluation of symmetry with the McWilliams runs test. Non-parametric data were Box-Cox transformed. Calculation of 95% reference limits was performed using the robust method for native or transformed normally distributed data sets, and the non-parametric method for non-Gaussian data sets. The 90% confidence interval (CI) of the limits was calculated using a non-parametric bootstrap method (34).

#### Clinical Validation and Evaluation of the Acute Phase Response

Results from the healthy group were compared with those from the injured group. Data sets with a normal distribution were compared using the independent *t*-test (for equal variances) or Welch test (for unequal variances). Variances were evaluated using an *F*-test. Data sets with a non-Gaussian distribution were compared using the Mann-Whitney test.

#### Diagnostic Utility

The sensitivity, specificity, and diagnostic accuracy of each assay for detecting inflammation (using the injured group to represent inflammation) was determined using receiver-operator characteristic (ROC) curve analysis, where the area under the curve (AUC) is a measure of diagnostic accuracy (35). The analyte concentration at the Youden index (differential positive rate or point on the ROC curve where both sensitivity and specificity are optimal) was also reported in order to determine whether a clinical decision limit would be more diagnostically useful than a reference limit for detecting inflammation (36). Stepwise logistic regression analysis was performed to evaluate the predictive value of using APR results in combination for detecting inflammation (4).

A *p* < 0.05 was used for all statistical tests apart from the Anderson-Darling test where *p* < 0.27 was used to increase specificity (37). Apart from reference interval generation, all data analyses were performed using MedCalc for Windows version 17.6 (MedCalc Software, Ostend, Belgium).

Ethics approval for this study was obtained from the University of Pretoria Animal Ethics Committee (certificate numbers V042-15, V011-17).

## Results

### Study Populations and Samples

The characteristics of both study populations have been fully reported elsewhere and can be viewed in Supplementary Table 1 (22). Briefly, the reference sample group consisted of 25 male and 25 female adult white rhinoceros. Fifty citrated plasma samples of adequate volume for fibrinogen determination and 50 serum samples with adequate volume for albumin, haptoglobin and iron determination were available; SAA analysis was carried out on only 28 of the 50 reference population samples, due to material constraints. The injured group consisted of 30 animals: five calves, two subadults and 23 adults. Sex distribution was 18 males and 12 females. Fourteen animals had bullet wounds only, three had fighting wounds, two had dehorning wounds, two had surgical wounds, two had wounds from bullets and other causes, and seven had wounds of unknown origin. The injuries were determined to be acute in 11 animals, chronic in 17 animals, and of unknown chronicity in the remaining two animals. Only nine citrated plasma samples were available for fibrinogen determination and only enough material for 28 SAA measurements from the injured group.

### Sample Analysis and Assay Performance

CV_I_ derived from internal quality control data were 2.7% for albumin, 4.2% for iron, and 7.6% for fibrinogen, which were within performance goals. Mean haptoglobin concentrations in the low and high concentration pools were 0.34 and 2.28 g/L, respectively. The haptoglobin assay had an intra-assay imprecision of 0.7%. Inter-assay imprecision, measured over two reagent kits (7 days for kit 1, 6 days for kit 2) was up to 30.6% (low pool) for the first kit and 19.4% (low pool) for the second kit. Linearity was tested up to 2.28 g/L and was acceptable [*r* = 1.0; slope and intercept (95% confidence intervals) of 1.0 (0.9 to 1.1) and 0.0 (−0.3 to 0.1)]. White rhinoceros samples with haptoglobin concentrations >2.28 g/L (the upper detection limit of the assay is 2.5 g/L) were subsequently diluted 1 in 3 with the calibrator diluent and re-assayed, as per the manufacturer's instructions.

The LoQ for the SAA ELISA was determined to be 20 mg/L, with imprecision of 49% for concentrations <20 mg/L and 6.7% above 20 mg/L. All results below 20 mg/L were therefore reported as “ <20 mg/L.” Linearity under dilution was tested from a blank sample up to 97 mg/L and was acceptable [*r* = 0.95; slope and intercept (95% confidence intervals) of 1.0 (0.9 to 1.1) and −6.1 (−20.7 to 8.4)]. SAA results higher than 97 mg/L were reported as “>97 mg/L.”

### Reference Intervals

SAA analysis was carried out on only 28 of the 50 samples, due to limited materials. Results from two individuals suspected to be dehydrated were excluded as albumin and iron were identified as high outliers using the Tukey test. One further outlier was identified and discarded for haptoglobin and fibrinogen (from the same animal). Three further outliers were determined by the Tukey test and visual examination in the remaining 26 results for SAA and eliminated. RIs are reported in Table 1 (individual results presented in Supplementary Table 1). All results for SAA were less than or equal to the LoQ and the RI for SAA is therefore reported as <20 mg/L.

**Table 1:** Reference intervals for acute phase reactants in the southern white rhinoceros, *Ceratotherium simum simum*.

**Measurands (Units)**	**Sample number**	**Mean**	**SD**	**Median**	**Min**	**Max**	**RI**	**LRL 90% CI**	**URL 90% CI**	**Distribution**	**Method**
Albumin (g/L)	48	25	3	24	18	31	18–31	17–19	30–32	G	R
Fibrinogen (g/L)	47	2.4	0.3	2.3	1.8	3.2	1.7–2.9	1.6–1.9	2.8–3.1^1^	G	R
Haptoglobin (g/L)	47	2.7	0.8	2.7	1.2	4.7	1.0–4.3	0.8–1.4	3.9–4.7^1^	G	R
Iron (μmol/L)	48	19.7	5.4	20.0	9.1	35.0	9.7–35.0	9.1–11.9	28.4–35.0^1^	NG	NP
Serum Amyloid A (mg/L)	23						<20^*^			NG	

*SD, standard deviation; RI, reference interval; CI, confidence interval; LRL, lower reference limit; URL, upper reference limit; G, Gaussian; NG, non-Gaussian; R, robust method; NP, non-parametric method*.

1 *CI of RL exceed RI by >20%*.

* *Functional sensitivity of assay 20 mg/L, all results <20 mg/L*.

### Clinical Validation and Diagnostic Utility

Individual results are presented in the Supplementary Table 1. The median (and ranges) for APRs in the injured group were: albumin 20 g/L (11–31 g/L); fibrinogen 2.9 g/L (2.4–3.3 g/L); haptoglobin 6.5 g/L (0.2–10.6 g/L); iron 7.9 μmol/L (2.9–48.5); SAA 31 mg/L (<20–97 mg/L). The haptoglobin measurements from the injured group included seven results below 2 g/L. Two of these were from animals with acute and five from animals with chronic injuries. Five of these animals (two acutely and three chronically injured) had increases in SAA above the reference interval. All seven samples were rerun at a 1 in 2 dilution; haptoglobin results did not differ.

**Figure 1:**
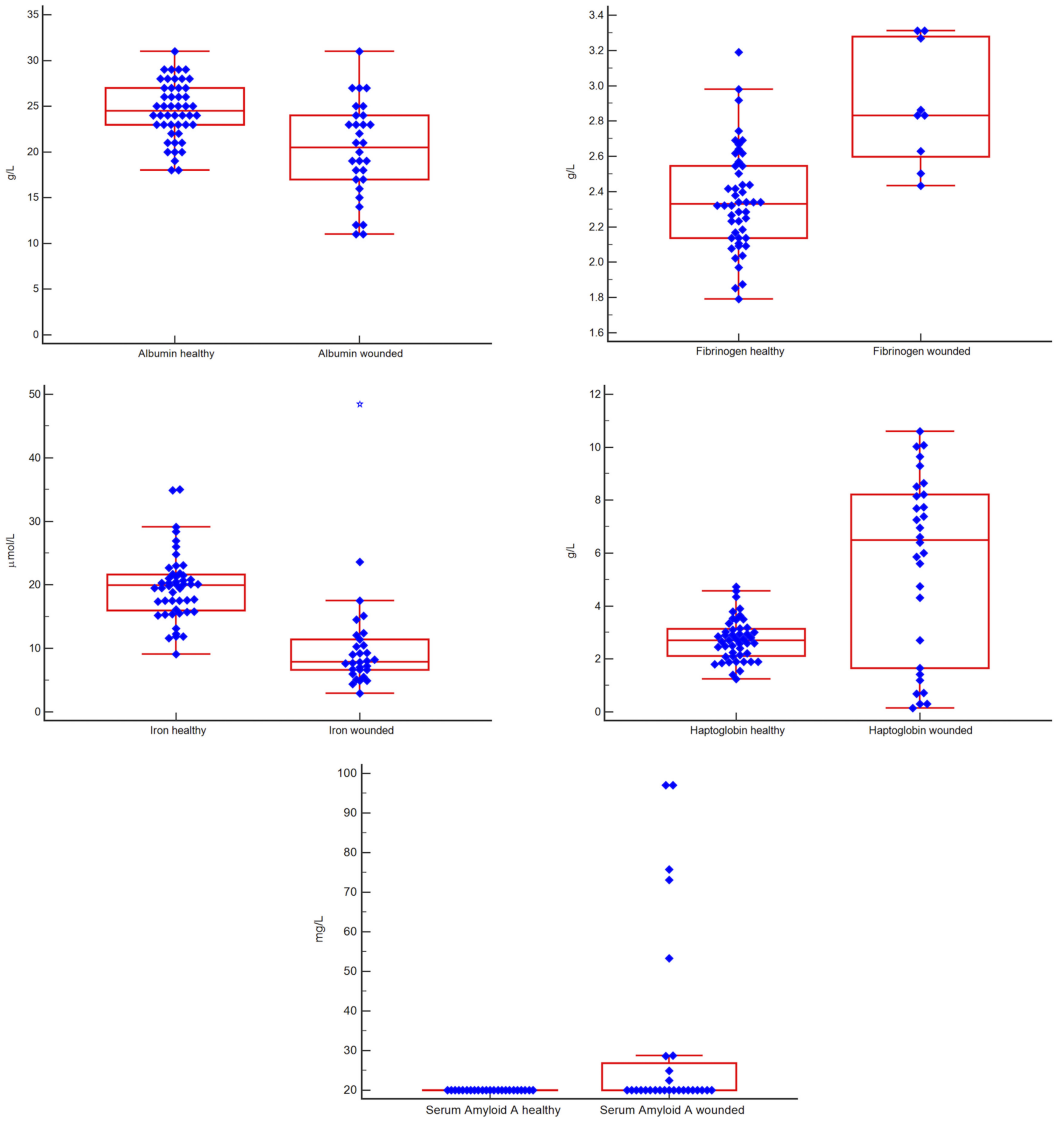
Box-and-whisker plots comparing results for five acute phase reactants in groups of healthy vs. injured white rhinoceros (*Ceratotherium simum simum)*. The blue diamonds indicate individual values, the central red box indicates the interquartile range, the central horizontal red line indicates the median, the outside horizontal red lines indicate minimum and maximum excluding “far out” values. A blue star represents a “far out” result (smaller/ larger than the lower/ upper quartile plus 3 times the interquartile range). There were significant differences (*p* < 0.0047) between the two groups for all measurands.

Albumin and iron were lower and fibrinogen up to 1.4-fold, haptoglobin up to 4-fold and SAA up to 5-fold higher in the injured vs. the healthy group (*p* < 0.0047 for all). These findings are shown in Figure 1.

**Figure 2:**
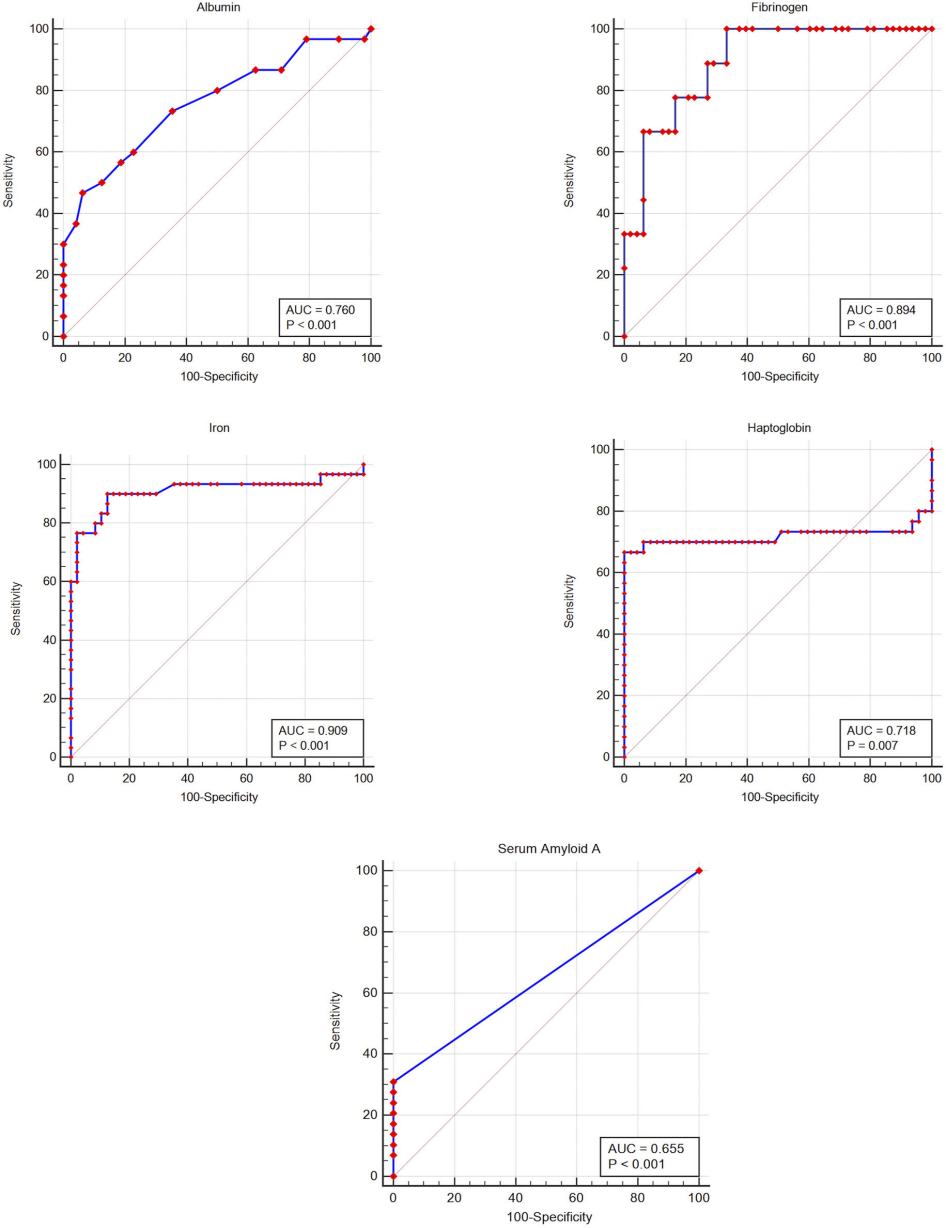
Receiver-operator characteristic curves for five acute phase reactants, for detecting inflammation in the white rhinoceros (*Ceratotherium simum simum)*. The gray line indicates the line of no discrimination; AUC, area under the curve.

The prevalence of inflammation (samples from injured animals compared to healthy), and diagnostic performance at the lower (albumin, iron) or upper (haptoglobin, SAA, fibrinogen) reference limit and at the Youden index cut-off (potential clinical decision limit) are shown in Table 2. Iron had a high and fibrinogen, albumin and haptoglobin a moderate diagnostic accuracy, according to the ROC curve analysis (Figure 2 and Table 2) (35). Although SAA showed high specificity, overall diagnostic accuracy was low due to the low sensitivity of this measurand (Figure 2 and Table 2).

**Table 2:** Indicators of diagnostic accuracy as determined by receiver-operator characteristic (ROC) curve analysis for five acute phase reactants, for differentiating healthy white rhinoceros (*Ceratotherium simum simum)* from those with inflammation.

**Measurand**	**Prevalence (%)^$^**	**Applicable LRL/URL**	**Se (%) at LRL/URL**	**Sp (%) at LRL/URL**	Clinical decision limit^*^	**Se (%) at clinical decision limit**	**Sp (%) at clinical decision limit**	**–LR at clinical decision limit**	**+LR at clinical decision limit**	**AUC**
Albumin (g/L)	38.5	18	36.7 (19.9–56.1)	95.8 (85.7–99.5)	≤ 19	46.7 (28.3–65.7)	93.75 (82.8–92.7)	0.57	7.47	0.76
Fibrinogen (g/L)	15.8	2.9	33.3 (7.5–70.1)	93.6 (82.8–98.7)	>2.4	100.0 (66.4–100.0)	66.7 (51.6–79.6)	0	3.0	0.89
Haptoglobin (g/L)	39.0	4.3	66.7 (47.2–82.7)	93.6 (82.5–98.7)	>4.7	66.7 (47.2–82.7)	100.00 (92.5–100.0)	0.33	∞	0.72
Iron (μmol/L)	38.5	9.7	60.0 (40.6–77.3)	97.9 (88.9–99.9)	≤ 15.1	90.0 (73.5–97.5)	87.5 (74.8–95.3)	0.11	7.2	0.91
Serum amyloid A (mg/L)	55.8	20	31.0 (15.3–50.8)	100.0 (84.6–100.0)	>20	31.0 (15.3–50.8)	100.0 (84.6–100.0)	0.69	∞	0.66

*95% confidence intervals for sensitivity and specificity are indicated in parentheses. LRL, lower reference limit; URL, upper reference limit; Se, sensitivity; Sp, specificity; –LR, negative likelihood ratio; +LR, positive likelihood ratio; AUC, area under the ROC curve*.

$ *Prevalence of inflammation based on number of samples analyzed*.

* *As indicated by the Youden index*.

Due to low sample numbers for SAA and fibrinogen, only albumin, haptoglobin, and iron were included in the logistic regression analysis. Albumin did not have a significant influence. Iron (odds ratio of 91.7 if <15.1 μmol/L) and haptoglobin (odds ratio of 101.3 if >4.7 g/L) were significant predictors of inflammatory status and when used together in parallel, predicted the clinical status of 91% of cases with a high diagnostic accuracy (ROC AUC of 0.96).

## Discussion

Most of the APR assays used in this study had an acceptable performance and were able to discriminate between white rhinoceros with and without inflammation with various degrees of diagnostic accuracy. Albumin and iron are negative APRs and haptoglobin, SAA and fibrinogen are positive APRs in this species.

Full validation of acute phase protein assays for most veterinary species is limited by the lack of a gold standard in the form of reference material or standards. However, acute phase protein assays aimed at measuring human proteins, or marketed for multispecies use, have been used in numerous wildlife species and shown to have cross-reactivity (8, 12, 38). Choice of reagent is important, as antibody-based assays do not show the same reactivity, resulting in different abilities to detect the measurand (e.g., SAA) and different reference intervals (7, 13, 38, 39). A modified approach to analytical validation of acute phase proteins conventionally includes demonstration of linearity (which is an indicator of accuracy), estimates of assay imprecision, and determination of differences in APR concentrations between a healthy and diseased group of animals known to have an inflammatory process (6, 8, 10, 12, 13, 40).

The intra-assay imprecision for haptoglobin was acceptable and similar to that reported for other non-domestic mammals (5, 8, 11, 38). The inter-assay imprecision was much higher (up to 30.6%) and exceeded the CV_MAX_ of 8.5%. Inter-assay imprecision for this assay in previous studies was mostly below 10%, apart from one finding of 19% in stellar sea lions (10, 16, 40–43). Most of these other studies performed the measurements for the inter-assay imprecision determination over 3 days, or do not mention the time interval. This experiment was performed twice for two reagent kits which were on-board the analyzer for 6–7 days which, although within the manufacturer's recommended maximum on-board time of 1 week, probably accounts for the high values. The impact on the results from healthy and injured animals was likely minimal though, as samples from each group of animals were batched and run within 48 h of opening each of the two kits and means from (the same) pools used to test imprecision on each kit were very similar. However, caution is advised if considering use of this assay over several days, at least on the Cobas Integra 400 Plus as the high imprecision may lead to clinically significant changes being missed, or non-clinically significant changes being misinterpreted.

The SAA ELISA test was chosen for use in this study as the automated immunoturbidometric assay routinely used in many veterinary laboratories does not appear to detect white rhinoceros SAA, probably due to lack of cross-reactivity of the antibodies in the latter with white rhinoceros SAA (44). Wide ranges of imprecision for this SAA ELISA have been reported for various species. For example, imprecision was up to 45% in studies using serum from dromedary camels (SAA results from 2.4 to 118.1 mg/L), 12.7% in goats (mean SAA 83.5 mg/L), 16.6% in cows (mean SAA 35 mg/L) and 37.5% in pigs (5.4 mg/L) (40, 42, 45, 46). These findings emphasize the importance of selecting a cut-off value (LoQ) below which the imprecision is unacceptably high and measured results cannot be reported as such. Modifying the calibration procedure to include lower concentration calibrators may improve precision at low SAA concentrations. Using a higher concentration high calibrator and modifying the dilution protocol would extend the linearity range to allow higher concentrations of SAA (above 97 mg/L) to be reported. These modifications are possible with this multispecies assay and should be attempted in future studies involving white rhinoceros.

The APR assays used in this study detected expected changes in the injured compared to the healthy group of white rhinoceros. However, several limitations exist with this clinical model. Firstly, the assumption that the reference sample group is healthy is based on observation and a basic clinical examination. For example, these animals were not parasite-free, as normal tick-loads were noted. However, some parasitism is considered normal in a free-ranging population, and white rhinoceros suffer from few inflammatory diseases. Chemical restraint is necessary for safe sampling of both healthy and injured white rhinoceros. The effect of immobilization would have been similar on both groups, and the short time involved in capture (typically 20 min from darting to sampling) is not enough to result in clinicopathological manifestations of an acute phase response (3). The reference values for APRs obtained here will still be useful for other white rhinoceros populations living under similar environmental and management conditions.

Since the injured white rhinoceros had varying severity and duration of wounds, an acute phase response may no longer have been present in those animals with less extensive or chronic wounds, and inflammation limited to the local area of trauma. Hematology was not available for most injured animals and leukocyte responses could not be evaluated. As mentioned previously, white rhinoceros do not commonly suffer from systemic inflammatory diseases and inducing inflammation experimentally would be considered unethical in this protected species. The clinical model for the acute phase response therefore has limitations, due to the heterogeneity of expected inflammation in this group. Another limitation is that the reference sample group consisted only of adults, while 7 of the 30 animals in the tissue-injury group were calves or subadults. Albumin and globulin concentrations are not different between white rhinoceros of different ages and so the effect of this heterogeneity is likely to be minimal (26).

As no other published studies document reference intervals for APRs in white rhinoceros, these results can be compared to those available for other Perissodactyla. The SAA RIs were 0.8–82.7 mg/L in black rhinoceros (same ELISA assay), 0.1–20.0 mg/L in horses and 1.8–31.4 mg/L in zebra (both immunoturbidometric assay) (6, 15, 47, 48). The SAA reference intervals in white rhinoceros are similar to those in horses and zebra, but lower than the black rhinoceros, which is a species predisposed to a pro-inflammatory state (15). Fibrinogen reference intervals for Perissodactyla, using the same modified Clauss method in this study, have not been published, but values in healthy horses appear to range from 2.0 to 3.5 g/L, similar to the reference interval in white rhinoceros (49–51). Similar to previous reports for plasma albumin and albumin determined by serum protein electrophoresis, serum albumin reference intervals are lower in the white rhinoceros than in other Perissodactyla species (22, 23). This may be due, in part, to the high globulin concentrations reported for this species (22). Serum iron RIs in white rhinoceros are slightly lower than in horses (14.3–43.0 μmol/L), which could be related to nutrition or the presence of low-grade inflammation in this population (4). The most striking difference, when comparing white rhinoceros to other species in this taxonomic order, was for haptoglobin, where reference intervals in this study were significantly higher than those reported for horses (0.29–2.26 g/L) and zebra (0.37–1.58 g/L) using the same assay (6, 52). The 90% CI of the upper reference limit exceeded the reference interval by 20% for iron, haptoglobin and fibrinogen, indicating that group sizes were too small to generate an accurate reference limit (32).

High normal haptoglobin concentrations in this reference sample group of white rhinoceros may reflect a high level of constitutive expression of this protein or the presence of chronic inflammation. This group also showed high resting α-2 globulin levels (16.1–26.6 g/L), documented in another study (22). Haptoglobin and other acute phase proteins like α-2-macroglobulin migrate to the α-2 fraction (2). Biological and immunomodulatory functions of haptoglobin include binding of hemoglobin and stabilization of iron, binding of nitrous oxide, upregulation of anti-inflammatory cytokines, downregulation of neutrophil function and suppression of T-lymphocytes (2). Increased haptoglobin in wildlife may reflect ongoing parasitic infection, association with environmental pollutants in marine mammals, and has been proposed as a marker for population health in non-domestic animal populations (14). For example, haptoglobin levels varied in Stellar sea lions and harbor seals from different marine locations, with higher values in areas where populations are declining, and in free-ranging vs. aquarium animals. These findings were thought to be due to different environmental stressors, diseases or genetic differences between geographically separated populations (17). Similarly, haptoglobin (but not SAA) was higher in free-ranging compared to captive Florida manatees, possibly due to occult inflammation and higher levels of cortisol from chronic stress, as production of haptoglobin and other acute phase proteins is also linked to activation of the hypothalamic-pituitary-adrenal axis (2, 53). Further studies on this population and other groups of white rhinoceros under different husbandry conditions would shed light on the reasons for the high haptoglobin concentration found here, and the utility of this marker for monitoring population health.

The finding of seven low haptoglobin results in injured animals is difficult to explain. Possibilities include a matrix or storage effect, or a prozone effect, although this concern was investigated further by repeating the analysis with a dilution. Only one dilution (1:2) was performed however, due to material constraints. It is also possible that these values truly represent low haptoglobin concentrations in these animals, although this is less likely, given that five had increased SAA.

Albumin and iron values were lower and fibrinogen, haptoglobin and SAA higher in injured white rhinoceros. These changes are consistent with expected values for negative and positive APRs and indicate that white rhinoceros have an acute phase response similar to that seen in horses and zebras, as well as various non-domesticated mammal species (5, 9, 11, 13, 15, 54). SAA is a major positive APP in most species and may increase up to 1,000 times above basal levels (55). The largest increase in this study was just under 5-fold, but accurate determination of very high and low SAA concentrations were limited by the set-up of the ELISA. Further optimization of the assay may reveal much higher increases compared to a lower baseline in future studies.

Using the reference intervals as a cut-off, all measurands generally had excellent specificity and poor to moderate sensitivity. This is expected when the same population that was used to generate reference intervals is used to examine diagnostic accuracy. Sensitivity improved and specificity decreased when using the Youden index-associated clinical decision limit for iron and fibrinogen, specificity improved for haptoglobin, and test characteristics did not significantly change for albumin. The cut-off and therefore the excellent specificity (100%) and poor sensitivity (32%) for SAA remained the same. This is different to a study in manatees where haptoglobin, fibrinogen and SAA were found to have excellent specificity (90–95%) but only SAA had good sensitivity (85%) for detecting disease, and in horses where SAA has both good specificity (up to 94%) and sensitivity (up to 82%) (4, 13, 47). The poor SAA sensitivity in this study was probably due to the fact that 17/30 animals had chronic injuries, and may no longer have a systemic inflammatory response. The four highest SAA concentrations were from the acute injury group, with only one result in the chronic injury group above 50 mg/L. Therefore, low SAA concentrations do not rule out the presence of chronic inflammation in this species. Iron had the best diagnostic accuracy, with high specificity and sensitivity at the clinical decision limit. The serum iron assay is commonly offered by reference laboratories and is therefore accessible to veterinarians.

The combination of decreased iron and increased haptoglobin had a higher diagnostic accuracy than any of the measurands alone. Fibrinogen and SAA were not included in the model because of low sample numbers, but since increased SAA had excellent specificity and fibrinogen ≤ 2.3 g/L excellent sensitivity, adding these two measurands to an APR profile would probably improve diagnostic accuracy.

The information gathered about APRs in this study may have use in monitoring the health of white rhinoceros populations, both free-ranging and captive. Changes in APRs in a population could indicate an increased incidence of disease or environmental stressors. An acute phase response in an individual animal could be useful for clinical diagnosis and monitoring, as in other species, and may prove helpful in the rehabilitation setting. More work needs to be done on optimizing assay performance, researching the differences between captive and free-ranging populations, and investigating changes in other diseases.

## Data Availability Statement

All datasets generated for this study are included in the article/Supplementary Material.

## Ethics Statement

The animal study was reviewed and approved by University of Pretoria Animal Ethics Committee. Written informed consent was obtained from the owners for the participation of their animals in this study.

## Author Contributions

EH contributed to conceptualization and study design, data curation, performed the data analysis, acquired funding, and wrote the first draft of the manuscript. MM contributed to conceptualization and design of the study and assisted with sample provision. CC, GS, and AG contributed to the conceptualization and design of the study. PB assisted with sample provision and data curation. MM, CC, GS, AG, and PB contributed to manuscript revision, read, and approved the submitted version.

### Conflict of Interest

The authors declare that the research was conducted in the absence of any commercial or financial relationships that could be construed as a potential conflict of interest.

## References

[1] Cray C Zaias J Altman NH. Acute phase response in animals: a review. Comp Med. (2009) 59:517–26. 20034426 PMC2798837

[2] Ceciliani F Ceron JJ Eckersall PD Sauerwein H. Acute phase proteins in ruminants. J Proteomics. (2012) 75:4207–31. 10.1016/j.jprot.2012.04.004 22521269

[3] Eckersall PD Bell R. Acute phase proteins: biomarkers of infection and inflammation in veterinary medicine. Vet J. (2010) 185:23–7. 10.1016/j.tvjl.2010.04.009 20621712

[4] Hooijberg EH van den Hoven R Tichy A Schwendenwein I. Diagnostic and predictive capability of routine laboratory tests for the diagnosis and staging of equine inflammatory disease. J Vet Intern Med. (2014) 28:1587–93. 10.1111/jvim.12404 25056342 PMC4895560

[5] Krogh AK Lundsgaard JF Bakker J Langermans JA Verreck FA Kjelgaard-Hansen M. Acute-phase responses in healthy and diseased Rhesus macaques (*Macaca mulatta*). J Zoo Wildl Med. (2014) 45:306–14. 10.1638/2013-0153R.1 25000691

[6] Cray C Hammond E Haefele H. Acute phase protein and protein electrophoresis values for captive Grant's zebra (*Equus burchelli*). J Zoo Wildl Med. (2013) 44:1107–10. 10.1638/2013-0033R.1 24450080

[7] Cray C Rodriguez M Dickey M Brewer LB Arheart KL Assessment of serum amyloid A levels in the rehabilitation setting in the Florida manatee (*Trichechus manatus* latirostris). J Zoo Wildl Med. (2013) 44:911–17. 10.1638/2012-0270R.1 24450049

[8] Isaza R Wiedner E Hiser S Cray C. Reference intervals for acute phase protein and serum protein electrophoresis values in captive Asian elephants (*Elephas maximus*). J Vet Diagn Invest. (2014) 26:616–21. 10.1177/1040638714543923 25057161

[9] Sheldon JD Johnson SP Hernandez JA Cray C Stacy NI. Acute-phase responses in healthy, malnourished, and otostrongylus-infected juvenile Northern elephant seals (*Mirounga angustirostris*). J Zoo Wildl Med. (2017) 48:767–75. 10.1638/2016-0267.1 28920814

[10] Stanton JJ Cray C Rodriguez M Arheart KL Ling PD Herron A. Acute phase protein expression during elephant endotheliotropic herpesvirus-1 viremia in Asian elephants (*Elephas maximus*). J Zoo Wildl Med. (2013) 44:605–12. 10.1638/2012-0174R1.1 24063088

[11] Bernal L Feser M Martinez-Subiela S Garcia-Martinez JD Ceron JJ Tecles F. Acute phase protein response in the capybara (*Hydrochoerus hydrochaeris*). J Wildl Dis. (2011) 47:829–35. 10.7589/0090-3558-47.4.829 22102653

[12] Bertelsen MF Kjelgaard-Hansen M Grøndahl C Heegaard PMH Jacobsen S. Identification of acute phase proteins and assays applicable in nondomesticated mammals. J Zoo Wildl Med. (2009) 40:199–203. 10.1638/2007-0125.1 19368263

[13] Harr K Harvey J Bonde R Murphy D Lowe M Menchaca M. Comparison of methods used to diagnose generalized inflammatory disease in manatees (*Trichechus manatus* latirostris). J Zoo Wildl Med. (2006) 37:151–9. 10.1638/05-023.1 17312794

[14] Kakuschke A Erbsloeh HB Griesel S Prange A. Acute phase protein haptoglobin in blood plasma samples of harbour seals (*Phoca vitulina*) of the Wadden Sea and of the isle Helgoland. Comp Biochem Physiol B. (2010) 155:67–71. 10.1016/j.cbpb.2009.10.002 19818410

[15] Schook MW Wildt DE Raghanti MA Wolfe BA Dennis PM. Increased inflammation and decreased insulin sensitivity indicate metabolic disturbances in zoo-managed compared to free-ranging black rhinoceros (*Diceros bicornis*). Gen Comp Endocrinol. (2015) 217–218:10–19. 10.1016/j.ygcen.2015.05.003 25980685

[16] Thomton JD Mellish J-AE. Haptoglobin concentrations in free-range and temporarily captive juvenile Steller sea lions. J Wildl Dis. (2007) 43:258–61. 10.7589/0090-3558-43.2.258 17495310

[17] Zenteno-Savin T Castellini MA Rea LD Fadely BS. Plasma haptoglobin levels in threatened Alaskan pinniped populations. J Wildl Dis. (1997) 33:64–71. 10.7589/0090-3558-33.1.64 9027692

[18] Borges AS Divers TJ Stokol T Mohammed OH. Serum iron and plasma fibrinogen concentrations as indicators of systemic inflammatory diseases in horses. J Vet Intern Med. (2007) 21:489–94. 10.1111/j.1939-1676.2007.tb02995.x 17552456

[19] Brosnahan MM Erb HN Perkins GA Divers TJ Borges AS Osterrieder N. Serum iron parameters and acute experimental EHV-1 infection in horses. J Vet Intern Med. (2012) 26:1232–5. 10.1111/j.1939-1676.2012.00963.x 22748124

[20] Modise A Minister Molewa Highlights Progress on Integrated Strategic Management of Rhinoceros. (2017) Available online at: https://www.environment.gov.za/mediarelease/molewa_progressonintegrated_strategicmanagement_ofrhinoceros (accessed August 2, 2017).

[21] Department of Environmental Affairs RSA Minister Edna Molewa Highlights Progress in the Fight Against Rhino Poaching. (2016) Available online at: https://www.environment.gov.za/mediarelease/molewa_highlightsprogress_onrhinopoaching2016 (accessed December 22, 2016).

[22] Hooijberg EH Miller M Cray C Buss P Steenkamp G Goddard A. Serum protein electrophoresis in healthy and injured southern white rhinoceros (*Ceratotherium simum simum*). PLoS ONE. (2018) 13:e0200347. 10.1371/journal.pone.0200347 30044807 PMC6059428

[23] Hooijberg EH Steenkamp G Buss P Goddard A. Method comparison and generation of plasma biochemistry RIs for the white rhinoceros on a point-of-care and wet chemistry analyzer. Vet Clin Pathol. (2017) 46:287–98. 10.1111/vcp.12490 28419525

[24] Hooijberg EH Steenkamp G du Preez JP Goddard A. Analytic and quality control validation and assessment of field performance of a point-of-care chemistry analyzer for use in the white rhinoceros. Vet Clin Pathol. (2017) 46:100–10. 10.1111/vcp.12456 28152184

[25] Pienaar DJ Hall-Martin AJ Hitchins PM Horn growth rates of free-ranging white and black rhinoceros. Koedoe. (1991) 34:97–105. 10.4102/koedoe.v34i2.426

[26] Mathebula N Miller M Buss P Joubert J Martin L Kruger M. Biochemical values in free-ranging white rhinoceros (*Ceratotherium simum.*) in Kruger National Park, South Africa. J Zoo Wildl Med. (2012) 43:530–8. 10.1638/2011-0259R.1 23082517

[27] Harr KE Flatland B Nabity M Freeman KP. ASVCP guidelines: allowable total error guidelines for biochemistry. Vet Clin Pathol. (2013) 42:424–36. 10.1111/vcp.12101 24320779

[28] Kjelgaard-Hansen M Mikkelsen LF Kristensen AT Jensen AL Study on biological variability of five acute-phase reactants in dogs. Comp Clin Path. (2003) 12:69–74. 10.1007/s00580-003-0477-z

[29] Flatland B Freeman KP Friedrichs KR Vap LM Getzy KM Evans EW. ASVCP quality assurance guidelines: control of general analytical factors in veterinary laboratories. Vet Clin Pathol. (2010) 39:264–77. 10.1111/j.1939-165X.2010.00251.x 21054473

[30] Hyslop NP White WH. Estimating precision using duplicate measurements. J Air Waste Manage Assoc. (2009) 59:1032–9. 10.3155/1047-3289.59.9.1032 19785269

[31] Westgard JO (Ed.). The detection limit experiment. Basic Method Validation, 3rd ed. Madison, WI: Westgard QC, Inc (2008). p. 168–85.

[32] Friedrichs KR Harr KE Freeman KP Szladovits B Walton RM Barnhart KF. ASVCP reference interval guidelines: determination of *de novo* reference intervals in veterinary species and other related topics. Vet Clin Pathol. (2012) 41:441–53. 10.1111/vcp.12006 23240820

[33] Geffré A Concordet D Braun J-P Trumel C. Reference value advisor: a new freeware set of macroinstructions to calculate reference intervals with Microsoft Excel. Vet Clin Pathol. (2011) 40:107–12. 10.1111/j.1939-165X.2011.00287.x 21366659

[34] Geffré A Braun JP Trumel C Concordet D. Estimation of reference intervals from small samples: an example using canine plasma creatinine. Vet Clin Pathol. (2009) 38:477–84. 10.1111/j.1939-165X.2009.00155.x 19473330

[35] Gardner IA Greiner M. Receiver-operating characteristic curves and likelihood ratios: improvements over traditional methods for the evaluation and application of veterinary clinical pathology tests. Vet Clin Pathol. (2006) 35:8–17. 10.1111/j.1939-165X.2006.tb00082.x 16511785

[36] Jensen AL Poulsen JSD. Evaluation of diagnostic tests using relative operating characteristic (ROC) curves and the differential positive rate. An example using the total serum bile acid concentration and the alanine aminotransferase activity in the diagnosis of canine hepatobiliary diseases. J Vet Med A. (1992) 39:656–68. 10.1111/j.1439-0442.1992.tb00231.x 1455934

[37] Le Boedec K. Sensitivity and specificity of normality tests and consequences on reference interval accuracy at small sample size: a computer-simulation study. Vet Clin Pathol. (2016) 45:648–56. 10.1111/vcp.12390 27556235

[38] Cray C Arheart KL Hunt M Clauss T Leppert LL Roberts K. Acute phase protein quantitation in serum samples from healthy Atlantic bottlenose dolphins (*Tursiops truncatus*). J Vet Diagn Invest. (2013) 25:107–11. 10.1177/1040638712467986 23242666

[39] Passler T Chamorro MF Riddell KP Edmondson MA van Santen E Cray C. Evaluation of methods to improve the diagnosis of systemic inflammation in alpacas. J Vet Intern Med. (2013) 27:970–6. 10.1111/jvim.12102 23662652

[40] Tecles F Fuentes P Martínez Subiela S Parra MD Muñoz A Cerón JJ. Analytical validation of commercially available methods for acute phase proteins quantification in pigs. Res Vet Sci. (2007) 83:133–9. 10.1016/j.rvsc.2006.10.005 17141287

[41] Martínez-Subiela S Cerón JJ Validation of commercial assays for the determination of haptoglobin, C-reactive protein and serum amyloid A in dogs. Arch Med Vet. (2005) 37:61–6. 10.4067/S0301-732X2005000100009

[42] Gonzalez FH Tecles F Martinez-Subiela S Tvarijonaviciute A Soler L Ceron JJ. Acute phase protein response in goats. J Vet Diagn Invest. (2008) 20:580–4. 10.1177/104063870802000507 18776089

[43] Pihl TH Andersen PH Kjelgaard-Hansen M Mørck NB Jacobsen S. Serum amyloid A and haptoglobin concentrations in serum and peritoneal fluid of healthy horses and horses with acute abdominal pain. Vet Clin Pathol. (2013) 42:177–83. 10.1111/vcp.12031 23577834

[44] Hooijberg EH Cray C Du Preez JP Steenkamp G Goddard A Routine inflammatory markers in an injured white rhinoceros (case report). Vet Clin Pathol. (2016) 45:E12 10.1111/vcp.12386

[45] Greunz EM Krogh AKH Pieters W Ruiz OA Bohner J Reckendorf A. The acute-phase and hemostatic response in dromedary camels (C*amelus dromedarius*). J Zoo Wildl Med. (2018) 49:361–70. 10.1638/2017-0221.1 29900796

[46] Eckersall PD Young FJ McComb C Hogarth CJ Safi S Weber A. Acute phase proteins in serum and milk from dairy cows with clinical mastitis. Vet Rec. (2001) 148:35–41. 10.1136/vr.148.2.35 11202551

[47] Belgrave RL Dickey MM Arheart KL Cray C. Assessment of serum amyloid A testing of horses and its clinical application in a specialized equine practice. J AmVet Med Assoc. (2013) 243:113–19. 10.2460/javma.243.1.113 23786199

[48] Jacobsen S Kjelgaard-Hansen M Hagbard Petersen H Jensen AL. Evaluation of a commercially available human serum amyloid A (SAA) turbidometric immunoassay for determination of equine SAA concentrations. Vet J. (2006) 172:315–19. 10.1016/j.tvjl.2005.04.021 15950503

[49] Andersen SA Petersen HH Ersbøll AK Falk-Rønne J Jacobsen S. Vaccination elicits a prominent acute phase response in horses. Vet J. (2012) 191:199–202. 10.1016/j.tvjl.2011.01.019 21371917

[50] Jacobsen S Jensen JC Frei S Jensen AL Thoefner MB. Use of serum amyloid A and other acute phase reactants to monitor the inflammatory response after castration in horses: a field study. Eq Vet J. (2005) 37:552–6. 10.2746/042516405775314853 16295934

[51] Jacobsen S Nielsen JV Kjelgaard-Hansen M Toelboell T Fjeldborg J Halling-Thomsen M. Acute phase response to surgery of varying intensity in horses: a preliminary study. Vet Surg. (2009) 38:762–9. 10.1111/j.1532-950X.2009.00564.x 19674420

[52] Cray C Belgrave RL Haptoglobin quantitation in serum samples from clinically normal and clinically abnormal horses. J Eq Vet Sci. (2014) 34:337–40. 10.1016/j.jevs.2013.05.007

[53] Harvey JW Harr KE Murphy D Walsh MT Chittick EJ Bonde RK. Clinical biochemistry in healthy manatees (*Trichechus manatus* latirostris). J Zoo Wildl Med. (2007) 38:269–79. 10.1638/1042-7260(2007)038[0269:CBIHMT]2.0.CO;2 17679511

[54] Depauw S Delanghe J Whitehouse-Tedd K Kjelgaard-Hansen M Christensen M Hesta M. Serum protein capillary electrophoresis and measurement of acute phase proteins in a captive cheetah (*Acinonyx jubatus*) population. J Zoo Wildl Med. (2014) 45:497–506. 10.1638/2013-0111R1.1 25314816

[55] Uhlar CM Whitehead AS. Serum amyloid A, the major vertebrate acute-phase reactant. Eur J Biochem. (1999) 265:501–23. 10.1046/j.1432-1327.1999.00657.x 10504381

